# Honey: A Promising Therapeutic Supplement for the Prevention and Management of Osteoporosis and Breast Cancer

**DOI:** 10.3390/antiox12030567

**Published:** 2023-02-24

**Authors:** Monika Martiniakova, Veronika Kovacova, Vladimira Mondockova, Nina Zemanova, Martina Babikova, Roman Biro, Sona Ciernikova, Radoslav Omelka

**Affiliations:** 1 Department of Zoology and Anthropology, Faculty of Natural Sciences and Informatics, Constantine the Philosopher University in Nitra, 949 01 Nitra, Slovakia; 2 Department of Botany and Genetics, Faculty of Natural Sciences and Informatics, Constantine the Philosopher University in Nitra, 949 01 Nitra, Slovakia; 3 Department of Genetics, Cancer Research Institute, Biomedical Research Center of Slovak Academy of Sciences, 845 05 Bratislava, Slovakia

**Keywords:** honey, osteoporosis, breast cancer, associations, preclinical studies, clinical trials, prevention, management, treatment

## Abstract

Osteoporosis and breast cancer are serious diseases that have become a significant socioeconomic burden. There are biochemical associations between the two disorders in terms of the amended function of estrogen, receptor activator of nuclear factor kappa beta ligand, oxidative stress, inflammation, and lipid accumulation. Honey as a functional food with high antioxidant and anti-inflammatory properties can contribute to the prevention of various diseases. Its health benefits are mainly related to the content of polyphenols. This review aims to summarize the current knowledge from in vitro, animal, and human studies on the use of honey as a potential therapeutic agent for osteoporosis and breast cancer. Preclinical studies have revealed a beneficial impact of honey on both bone health (microstructure, strength, oxidative stress) and breast tissue health (breast cancer cell proliferation and apoptosis, tumor growth rate, and volume). The limited number of clinical trials, especially in osteoporosis, indicates the need for further research to evaluate the potential benefits of honey in the treatment. Clinical studies related to breast cancer have revealed that honey is effective in increasing blood cell counts, interleukin-3 levels, and quality of life. In summary, honey may serve as a prospective therapeutic supplement for bone and breast tissue health.

## 1. Introduction

Menopause is a biological process characterized by dysfunction of ovarian follicles and estrogen deficiency, oxidative stress, and inflammation, that together lead to different chronic disorders [1,2]. When the organism is exposed to high levels of oxidative stress following estrogen depletion, lipid accumulation also occurs [3]. Osteoporosis and breast cancer are considered serious diseases in which the aforementioned factors are involved and are currently becoming a significant socioeconomic burden worldwide.

Generally, postmenopausal osteoporosis is characterized by reduced bone mineral density (BMD) and increased risk of fragility fractures that are associated with significant pain, suffering, and disability [4]. Moreover, hip and vertebral fractures are consistent with significantly increased mortality [5]. It has been reported that a decrease in estrogen production represents a major cause of reduced bone mass [6,7,8]. During menopause, the osteoprotective effect of estrogen is weakened, leading to elevated expression of pro-inflammatory cytokines that promote osteoclastogenesis [9,10,11,12]. In general, estrogen regulates bone metabolism through two receptors: estrogen receptor alpha (ERα) and estrogen receptor beta (ERβ), with ERα being more dominant. Loss of estrogen also influences osteoblast progenitor cells via reduced ERα expression and lower responsiveness to mechanical stimulation [13]. Thus, estrogen deficiency not only directly affects the differentiation of precursor cells more toward active osteoclasts and less toward osteoblasts but can also influence their cellular energetics. Increased adiposity and inflammation after menopause can indirectly lead to bone loss as well [14].

Similar to bones, breast tissue is also dependent on estrogen [15]. In breast carcinogenesis, elevated exposure to estrogen is linked with early menarche, late menopause, obesity, and estrogen replacement therapy. High blood estrogen levels are able to increase the risk, incidence, and severity of breast malignancy in pre- and postmenopausal women [16]. In general, breast cancer is the second leading cause of cancer death in women, with a higher prevalence in postmenopausal women [1,17]. Consequently, postmenopausal women are at risk of morbidity and mortality, which are a combination of both diseases mentioned above.

Current pharmacological treatment for osteoporosis and breast cancer is often associated with adverse side effects; therefore, various natural therapeutic substances have been intensively studied to find an alternative and effective treatment method with less harmful impacts [17,18]. Honey and other bee products (e.g., royal jelly, propolis, bee bread, drone brood homogenate) are widely used as a functional food due to their high antioxidant and anti-inflammatory properties, which contribute to the prevention of various diseases, including diabetes, osteoporosis, cancer, reproductive disorders [19,20,21,22,23].

In general, honey is a sweet viscous liquid stored in combs after bees collect it from plants. It is produced by regurgitation, enzymatic activity, and evaporation of water in the hives. In addition to the source of nectar, bees also collect insect secretions (belonging to the genus *Rhynchota*) to produce honeydew honey [2,24,25]. Honey consists of at least 181 substances, mainly carbohydrates such as fructose (38%) and glucose (31%). It also contains enzymes, proteins, amino acids, polyphenols, vitamins, and minerals in lower quantities [26]. The content of polyphenols, which cover a wide spectrum of phytochemicals and are found in almost all types of natural honey, contributes to its health-promoting potential. Such polyphenols include flavonoids (e.g., quercetin, kaempferol, luteolin, hesperetin, chrysin, apigenin, galangin), phenolic acids (e.g., ellagic, caffeic, gallic, ferulic, benzoic, ascorbic), antioxidant enzymes (e.g., catalase, glucose oxidase, peroxidase) and carotenoids [27,28]. Most of these compounds interact with each other to create a range of synergistic antioxidant properties. Many studies revealed antioxidant, antibacterial, antiviral, immunomodulatory, anti-inflammatory, hypocholesterolemic, hypotensive, and antitumor impacts of honey [2,29]. The composition of a particular honey sample depends to a large extent on the nectar composition, the method of nectar collecting, environmental and seasonal factors, geographical origin, as well as storage conditions [30].

This review aims to summarize the current knowledge from preclinical and clinical studies regarding the use of honey as a potential therapeutic agent for osteoporosis and breast cancer due to their elevated incidence in postmenopausal women. Biochemical connections between the two disorders are also provided.

## 2. Biochemical Associations between Osteoporosis and Breast Cancer

**Figure 1:**
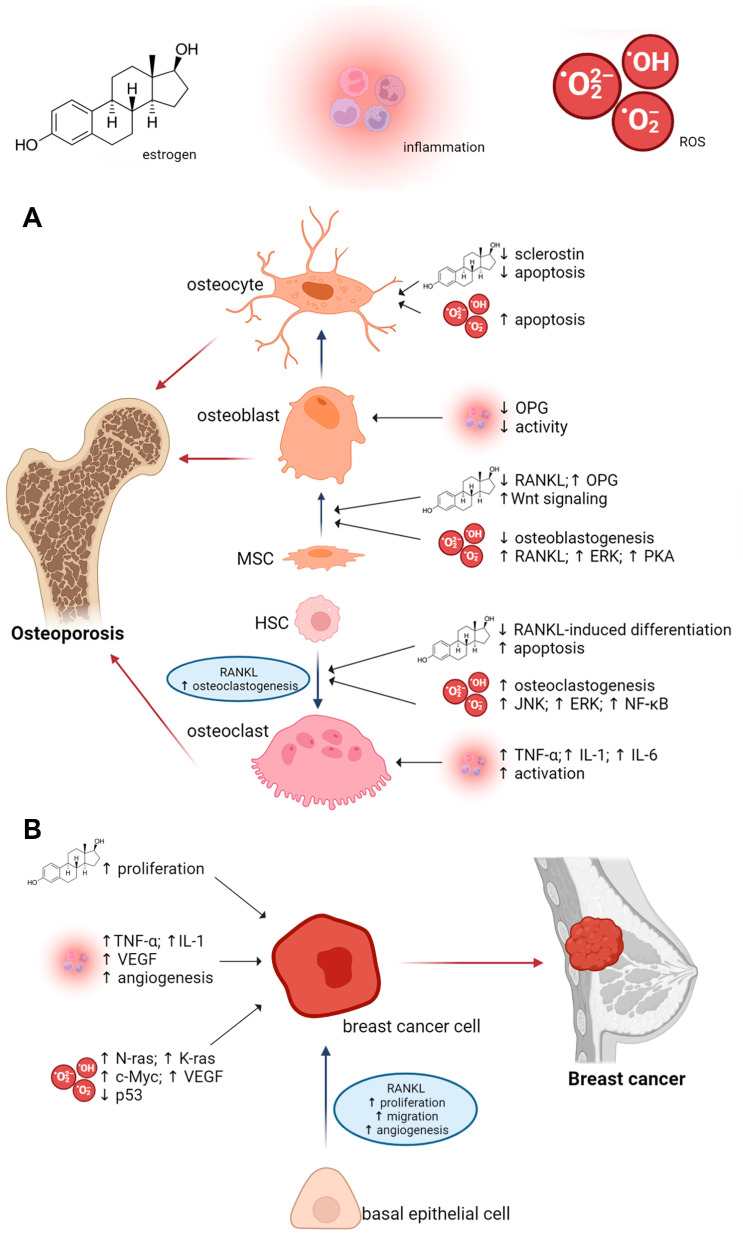
The impact of RANKL, estrogen, ROS, and inflammation on the development of osteoporosis (**A**) and breast cancer (**B**) (created with BioRender.com, https://www.biorender.com/, accessed on 27 January 2023). Abbreviations: c-Myc—c-myelocytomatosis oncogene product; ERK—extracellular signal-regulated kinase; HSC—hematopoietic stem cell; IL-1—interleukin 1; IL-6—interleukin 6; JNK—c-Jun N-terminal kinase; K-ras—Kirsten rat sarcoma viral oncogene homolog; MSC—mesenchymal stem cell; N-ras—neuroblastoma RAS viral oncogene homolog; NF-κB—nuclear factor kappa-B; OPG—osteoprotegerin; p53—tumor protein p53; PKA—protein kinase A; RANKL—receptor activator of nuclear factor kappa-B ligand; ROS—reactive oxygen species; TNF-α—tumor necrosis factor-alpha; VEGF—vascular endothelial growth factor; ↑—increased; ↓—decreased.

Biochemical connections between osteoporosis and breast cancer include the amended function of receptor activator of nuclear factor kappa beta ligand (RANKL), estrogen, reactive oxygen species (ROS)-induced oxidative stress, chronic low-grade inflammation, and lipid accumulation [1,2]. A clearer understanding of the associations between these diseases can lead to the development of a therapeutic target for postmenopausal breast cancer patients. Figure 1 illustrates the influence of RANKL, estrogen, ROS, and inflammation on the development of osteoporosis and breast cancer.

RANKL is an important cytokine that is a member of the tumor necrosis factor (TNF) family and is encoded by the tumor necrosis factor ligand super family 11 (*TNFSF11*) gene [31]. It plays an important role in human physiology by controlling the differentiation and activation of osteoclasts [32]. Generally, RANKL binds to the receptor activator of nuclear factor kappa beta (RANK) on osteoclast precursor cells. RANKL/RANK interaction subsequently activates nuclear factor kΒ (NF-kB) and supports the expression of other osteoclastogenic factors. Conversely, a soluble decoy receptor for RANKL-osteoprotegerin (OPG) prevents RANKL from binding to RANK. Therefore, RANKL/RANK/OPG system is considered a key mediator of osteoclastogenesis [18,33]. Moreover, RANKL/RANK pathway has been implicated in breast development as well as breast carcinogenesis. According to Fata et al. [34], lactating mammary gland did not develop cancer in RANK and RANKL receptor-deficient mice. In the study by Gonzalez-Suarez et al. [35], the development of mammary carcinogenesis was related to a higher expression of RANKL in 7,12-dimethylbenzeneanthracene (DMBA)-induced mice, with accelerated breast carcinogenesis identified in RANK-transgenic mice. RANKL also initiates the formation of pre-cancerous lesions and the metastatic process. Additionally, RANKL up-regulates the angiogenic process by stimulating the proliferation and survival of endothelial cells (Figure 1).

Both bone and breast tissues are dependent on estrogen. Moreover, high BMD can be associated with the risk of breast cancer [36]. The hormone estrogen is a key regulator of BMD [37], maintaining the balance between bone formation and bone resorption [38]. Specifically, estrogens stimulate osteoblast differentiation and activate Wnt signaling. They also have an indirect effect through suppression of RANKL and up-regulation of OPG, which ultimately inhibits osteoclastogenesis. Another mechanism for preventing bone resorption is the induction of apoptosis in osteoclasts. Furthermore, estrogens act at the osteocyte level since estrogen decreases sclerostin level and osteocyte apoptosis (Figure 1). Epidemiological and clinical evidence has shown that factors consistent with raised estrogen levels during a woman’s lifetime (e.g., early menarche, late menopause, late first full-term pregnancy, obesity) are related to increased risk of breast cancer [39]. Estrogens are generally believed to induce breast cancer cell proliferation via the ER and serve as a transcription factor to regulate the expression of target genes encoding proteins with important biological functions [40]. The impact of estrogen on both aforementioned diseases documents the fact that women who develop ER-positive breast cancer at a relatively younger age and are treated with anti-estrogen drugs such as tamoxifen have an elevated risk of postmenopausal osteoporosis [41]. Due to the role of estrogen in breast cancer, aromatase inhibitors (inhibitors of the estrogen-metabolizing enzyme aromatase) are used in the treatment of postmenopausal individuals with ER-positive breast cancer, despite problems with bone fractures. Recently, researchers are examining the potential of denosumab, an anti-RANKL antibody, in preventing aromatase inhibitor-associated bone loss [42], which could provide major benefits for postmenopausal breast cancer patients.

Oxidative stress is a contributing factor in many chronic diseases, including osteoporosis and breast cancer [43,44]. ROS directly promote osteoclast formation in a process mediated by RANKL-RANK interaction and enhance bone resorption [45,46]. This signaling pathway includes redox-sensitive components such as tumor necrosis factor receptor-associated factor 6 (TRAF6), Rac1 (a member of the Rho-GTPase subfamily), and nicotinamide adenine dinucleotide phosphate oxidases (NOX) [47]. Moreover, ROS induce apoptosis of osteoblasts and osteocytes by activating numerous signaling pathways. Mitogen-activated protein kinases such as ERK and JNK are involved in this process (Figure 1). ROS also reduce osteoblast activity and differentiation, thus mineralization and osteogenesis [48]. Postmenopausal women are not only exposed to high levels of oxidative stress, but also to elevated levels of nitric oxide (NO) in erythrocytes [49]. NO can increase the ability of cytokines to stimulate osteoclast activity and enhances their inhibitory impacts on osteoblast growth [50,51]. Ultimately, bone formation prevails over bone resorption. In breast cancer, oxidative stress has been implicated in the initiation, promotion, and progression grades of breast carcinogenesis [52]. Mammary tissue is a complex combination of different cell types, including stromal and neoplastic cells [53]. In cancerous breast tissue, stromal fibroblasts acquire a phenotype characterized by raised levels of cytokines, growth factors, and metalloproteinases [54]. In the tumor microenvironment, an altered redox state in favor of pro-oxidants induces the formation of activated fibroblasts, leading to modifications of epithelial cells that support tumorigenesis [55]. Oxidative stress in the tumor microenvironment is also characterized by activated stromal cells that generate tumor-enhancing signals, thereby promoting tumor growth and vascularization [56]. Elevated ROS induce oncogenes and DNA damage, inhibit tumor suppressor genes, and can interfere with different signaling pathways (Figure 1).

Chronic age-related inflammation also plays an important role in the pathogenesis of osteoporosis by affecting bone remodeling [57]. In the presence of RANKL, pro-inflammatory cytokines such as tumor necrosis factor alpha (TNF-α), interleukins (IL)-1, and IL-6 cause the excessive formation of osteoclasts and simultaneously inhibit the activities of osteoblasts [58]. The aforementioned cytokines also stimulate osteoclast development and elevate the production of macrophage colony-stimulating factor (M-CSF) by bone marrow stromal cells (BMSC) [59,60]. They also suppress osteoblasts in releasing OPG [58]. According to several studies, raised levels of cytokine-mediated acute phase C-reactive protein [61], and pro-inflammatory cytokines, including IL-6 [62], IL-1β [63], and TNF- α [64] are found in breast cancer patients, documenting that breast cancer is associated with inflammation. Elevation of these cytokines has been linked with breast cancer invasiveness and has also been used as a prognostic factor in breast cancer patients [65]. Inflammatory cells such as macrophages play a role during tumor progression by stimulating angiogenesis via the production of pro-inflammatory cytokines and VEGF (Figure 1).

Obesity, one of the abnormalities of lipid metabolism, has been hypothesized to protect the skeleton by increasing BMD [1] through mechanical loading, which stimulates bone formation by reducing apoptosis and increasing the proliferation and differentiation of osteoblasts and osteocytes [66,67]. This mechanism is supposed to be controlled by the Wnt/β-catenin signaling pathway [68,69]. For this reason, bone mass increases as a compensatory mechanism to adapt to a greater load [70]. However, several researchers reported conflicting findings. According to Hsu et al. [71] and Pollock et al. [72], excess fat mass was associated with reduced total BMD and total bone mineral content. The link between obesity and 13 cancer types, including ER-positive postmenopausal breast cancer, was established by International Agency for Cancer Research [73]. Moreover, obesity was consistent with poor response outcomes in patients with ER-positive breast cancer [74]. Therefore, obesity presents a challenge in treating individuals with postmenopausal breast cancer who suffer from osteoporosis [75]. Targeting the metabolic pathways linked to estrogen production and immune surveillance modulation might represent an effective trend in breast cancer prevention and treatment [76]. Studies on estradiol depletion by aromatase inhibitors in subjects with postmenopausal breast cancer indicate that higher levels of aromatase activity associated with elevated adipose tissue mass, reduce the efficacy of aromatase inhibitor therapy [77]. Nowadays, bisphosphonates are used to prevent aromatase inhibitor-induced bone loss and improve survival in postmenopausal patients with ER-positive breast cancer [78,79].

## 3. Honey and Osteoporosis

Honey is able to protect the bone mainly due to antioxidant and anti-inflammatory properties, primarily through its content of polyphenols, which act on several signaling pathways, resulting in anabolic and antiresorptive effects [2]. From the group of polyphenols, the anti-osteoporotic impact of quercetin, kaempferol, and luteolin was recorded [4]. In addition, vitamin D3 and its hydroxyderivatives with antioxidant properties were also detected in honey [80,81]. Vitamin D3 supplementation was found to have protective effects on the inhibition of bone loss and BMD in both experimental animals and postmenopausal women [82,83,84].

According to Zaid et al. [85], the thickness of trabecular bone was elevated in ovariectomized (OVX) rats receiving Tualang honey (a type of Malaysian honey that is especially produced by the rock bee) at the dose of 0.2 g/kg/day for 2 weeks compared to OVX rats fed calcium [86]. Additionally, identical Tualang honey administration (0.2 g/kg/day for 2 weeks) significantly increased BMD in OVX rats [87]. The study of Husniati Y et al. [88] showed that daily consumption of Tualang honey (20 mg/day for 4 months) in postmenopausal women resulted in similar bone densitometry findings as in individuals with hormone replacement therapy. Moreover, Shafin et al. [89] revealed that postmenopausal women consuming Tualang honey (20 g for 16 weeks) had comparable blood oxidative stress (e.g., glutathione peroxidase, catalase, superoxide dismutase) levels to those receiving estrogen-progestin therapy. The aforementioned beneficial effects of Tualang honey can be attributed to the highest content of kaempferol, quercetin, ellagic acid, gallic acid, hesperetin, and catechin among different types of honey, indicating its highest antioxidant potential [90,91,92].

According to Kamaruzzaman et al. [93], the administration of Kelulut honey (a type of Malaysian honey that is mainly produced by stingless bumblebees) at doses of 200 mg/kg/day and 400 mg/kg/day for 2 months alleviated glucocorticoid-induced osteoporosis through its antioxidant activity in rats. Significantly elevated bone volume/tissue volume, trabecular number, osteoblast surface, superoxide dismutase level and decreased osteoclast surface and malondialdehyde levels were determined in osteoporotic rats fed this type of honey. The impact of Kelulut honey supplementation (1 g/kg for 8 weeks) on the bone health of rats with metabolic syndrome was investigated by Ekeuku et al. [94]. Oxidative stress and chronic low-grade inflammation present in metabolic syndrome are known to play a major role in osteoporosis induction or bone loss [19]. Rats receiving Kelulut honey showed a significant reduction in osteoclast surface compared to the control group, other bone parameters did not differ between the two groups [94]. However, Ramli et al. [24] report that honey has a strong potential to be used in the management of metabolic syndrome and related osteoporosis by exerting anti-obesity, hypolipidemic, antidiabetic, and hypotensive activities.

Yudaniayanti et al. [30] examined the impact of honey supplements on bone strength in OVX rats. These authors determined significantly increased bone strength in OVX rats receiving honey (2 g/kg and 4 g/kg for 12 weeks) in comparison with the untreated group. According to Hasib et al. [95], honey administration (1 g/kg, 2 g/kg, and 4 g/kg for 2 weeks) had a favorable effect on osteoporotic fracture healing in rat femur by promoting osteoblastogenesis. The pro-osteoblastic influence of honey was documented by an enhanced level of alkaline phosphatase (ALP) in the serum.

Abu-Serie et al. [96] revealed the ameliorative impact of a combined extract of Greek thyme (*Thymus vulgaris*) and honey on hydrocortisone-induced osteoporosis in rat bone cells through modulation of bone turnover, oxidative stress, and inflammation. Moreover, a stronger anti-osteoporotic effect of the combined extract was recorded compared to a commonly used bisphosphonate drug (alendronate).

Interestingly, Manuka honey (a type of New Zealand honey with antimicrobial and antioxidant capacities) was used as an antibacterial agent incorporated into a biopolymer coating based on corn protein zein to evaluate the combined effects of bioactive glass and Manuka honey in a new type of scaffold. According to the results of Arango-Ospina et al. [97], Manuka honey and zein coatings imparted antibacterial properties and excellent mechanical properties to bioactive glass bone tissue scaffolds.

From the information mentioned above it is clear that honey may serve as a promising therapeutic supplement for the prevention and management of osteoporosis. Anyway, more scientific or epidemiological evidence is needed for the use of any type of honey in the treatment of postmenopausal osteoporosis in women due to the limited number of clinical trials. Summary data from the aforementioned research is presented in Table 1.

## 4. Honey and Breast Cancer

Honey as a potential preventive and therapeutic supplement is currently gaining attention in cancer research. Various studies have been reported to investigate the anticancer benefits of different types of honey from different origins. The anticancer activity of honey has been demonstrated against various cancer cell lines and tissues, such as breast, prostate, colorectal, endometrial, and renal [98,99,100,101,102,103,104,105,106]. In general, the chemo-preventive properties of honey are consistent with its bioactive compounds, mostly quercetin, luteolin, chrysin, and esters of caffeic [107]. Although the exact mechanism is still unclear, some studies revealed the interference of bioactive compounds with anti-proliferative [108], antioxidant [109], and pro-apoptotic cell-signaling pathways [110]. Choi et al. [111] documented the anti-proliferative effects of quercetin in the human breast cancer cell line MCF-7 by inhibiting cell cycle progression via transient accumulation in the M phase followed by G2 arrest. Moreover, quercetin treatment activated apoptosis in MCF-7 cells via the p38MAPK signaling pathway [112]. Kim et al. [113] detected melatonin and its metabolites in honey, which possess strong free radical scavenging properties [114]. However, high concentrations of melatonin can induce the production of ROS, leading to apoptosis in a variety of cancers [115,116,117]. In general, favorable impacts of honey against breast cancer have been proven in both preclinical and clinical studies.

Tualang honey has been found to induce apoptosis of MDA-MB-231 and MCF-7 breast cancer cells through activation of the mitochondrial apoptotic pathway by elevating caspase-3/7 and caspase-9 and reducing mitochondrial membrane potential [102]. Moreover, Tualang honey combined with tamoxifen enhanced the anticancer activity of tamoxifen, activated multiple caspase enzymes, and increased mitochondrial membrane depolarization, leading to a breast cancer cell (MCF-7 and MDA-MB-231) apoptosis [118]. Tualang honey with tamoxifen can therefore be used as an alternative for the treatment of breast cancer, thereby reducing the required dose of tamoxifen and subsequently eliminating the side effects of tamoxifen. According to Kadir et al. [119], the growth of DMBA-induced mammary tumors was inhibited by Tualang honey administration (0.2–2 g/kg for 150 days) in rats. Additionally, vascular endothelial growth factor (VEGF), a pro-angiogenic factor, was reduced in honey-supplemented rats. In the study of Zakaria et al. [120], elevated levels of alanine aminotransferase were determined in postmenopausal women with breast cancer compared to those consuming Tualang honey (20 g/day for 12 weeks). Moreover, an increase in creatinine levels, leukocyte, and platelet counts was observed in the honey-treated group. In a clinical trial by Hizan et al. [121], the combination of Tualang honey with the aromatase inhibitor anastrozole lowered background parenchyma enhancement (a correlate of cancer relapse) more efficiently than anastrozole treatment alone (42% vs. 10% reduction) in patients with ER-positive breast cancer.

The anti-proliferative impact of Manuka honey was determined in MDA-MB-231 and MCF-7 breast cancer cells and was time- and dose-dependent. Moreover, the IL-6/STAT3 signaling pathway was highlighted as one of the first potential targets for Manuka honey-induced breast cancer cell suppression [122]. In another study, Aryappalli et al. [123] found that inhibition of tyrosine-phosphorylated STAT3 in breast cancer cells by Manuka honey is mediated by selective antagonism of the IL-6 receptor. Ahmed et al. [124] revealed that supplementation with both Manuka and Tualang kinds of honey (1.0 g/kg for 120 days) was able to reduce tumor volume, numbers, weight, and growth rate in the 1-methyl-1-nitrosourea (MNU)-induced breast cancer in rats. In addition, a higher expression of pro-apoptotic proteins and lowered expression of anti-apoptotic proteins were recorded. These types of honey administered orally exhibit anticancer effects by modulating the immune system and activating the intrinsic apoptotic pathway.

Greek honey extract (pine, thyme, and fir) reduced the viability of MCF-7 breast cancer cells [100,125], while thyme honey inhibited the progression of MCF-7 cells by suppressing estrogenic impacts [100]. Anatolian honey with different botanical origins (pine, chestnut, and cedar) produced stronger inhibitory effects on MDA-MB-231, MCF-7, and SKBR3 breast cancer cells in a time- and dose-dependent manner [126]. In MCF-7 and MDA-MB-231 cancer cells, the aforementioned types of honey suppressed breast cancer through the IL-6/STAT3 signaling pathway.

Kurniawan et al. [127] examined the impact of apis Dorsata honey (two tablespoons orally, 3 times/day for 15 days) on IL-3 (multi-potential hematopoietic growth factor) levels in breast cancer patients undergoing chemotherapy. These authors determined increased levels of IL-3 in the honey-treated group compared to the control group. The effect of Dorsata honey on IL-6 (breast cancer metastases factor) levels and T lymphocytes in post-chemotherapy breast cancer individuals was investigated by Syam et al. [107]. It has been found that there is a significant increase in the levels of T lymphocytes, which can indirectly enhance the immune system and inhibit tumor cell growth in honey-treated patients with breast cancer. The results also showed that Dorsata honey consumption did not affect IL-6 levels in contrast to the Manuka honey, where differences were noted.

The ability of honey to mitigate the chemo- and radiotherapy-induced oral mucositis (OM) was documented in numerous studies that mainly involved patients with head and neck cancers [128]. The studies on honey-treatment toxicity associations are limited in breast cancer but a pilot randomized trial comprising breast cancer patients receiving doxorubicin and cyclophosphamide reported the clinical efficacy of propolis plus bicarbonate in OM prevention [129]. According to Aghamohammadi et al. [130], a mixture of honey (30 g) and cinnamon (4 g) powder administered to breast cancer patients three times a day for 1 week led to a significant improvement in overall health and quality of life after the treatment.

Although honey supplementation has been associated with breast cancer modification in most of the experimental studies mentioned above, further experiments (especially animal studies and prospective randomized clinical trials) are still needed to evaluate the potential usefulness of honey as a therapeutic supplement in prevention and management of breast cancer. Table 2 provides summary data from the aforementioned studies.

## 5. Conclusions

Nowadays, the administration of dietary supplements and functional food intake in standard care of osteoporotic and oncological patients is gaining more attention. Honey is one of the oldest organic natural substances used for medical purposes. Many studies have pointed to the antioxidant, antibacterial, antiviral, immunomodulatory, anti-inflammatory, hypocholesterolemic, hypotensive, and antitumor impacts of honey, making it beneficial for human health.

In this review, the current knowledge from in vitro, animal, and human studies concerning the use of honey as a potential therapeutic supplement for osteoporosis and breast cancer is presented, due to their increasing incidence in postmenopausal women. Preclinical studies related to osteoporosis have reported favorable effects of honey on cortical and trabecular bone microstructure, bone strength, and oxidative stress. The limited number of clinical trials suggests the need for further research to evaluate the potential benefits of honey in the treatment of postmenopausal osteoporosis. In relation to breast cancer, in vitro experiments revealed the anti-proliferative and pro-apoptotic impact of honey on breast cancer cells, as well as their increased apoptosis. Animal studies have shown that honey reduces the number, growth rate, volume, and tumor weight. Findings from clinical trials reported its immunomodulatory properties showing that honey is effective in increasing leukocyte and platelet counts, IL-3 levels, and quality of life. In this context, the potential role of honey and its oligosaccharides as prebiotics for specific beneficial bacteria might be examined in future clinical studies.

In conclusion, we can state that honey represents a prospective therapeutic supplement for bone and breast tissue health. However, several issues need to be addressed before administration, including the presence of allergens or pesticides, antibiotics, and contaminants. Since the existing differences among honey types, precise identification and quantification of bioactive compound content should be provided in detail. In addition, clinical studies published so far are limited by a small sample size without the involvement of all different ethnicities, a single dose of honey and often a short duration of experiments and different parameters analyzed. Therefore, further clinical trials should also be aimed at eliminating these shortcomings. Importantly, large-scale placebo-controlled clinical studies concerning nutrigenomics are highly warranted to evaluate the effects of honey with its bioactive components on global gene and protein expression.

## Figures and Tables

**Table 1:** Preclinical and clinical studies on the anti-osteoporotic potential of honey.

Research Models	Applied Treatment	Obtained Results	References
OVX Rats	Tualang honey; 0.2 g/kg/day/2 weeks	↑BV/TV ↑Tb.Th ↑Tb.N ↓Tb.Sp	[85]
OVX Rats	Tualang honey; 0.2, 1.0, and 2.0 g/kg/2 weeks	Tibia: ↑BMD	[87]
OVX Rats	Apis dorsata honey; 2 and 4 g/kg/12 weeks	↑Bone strength	[30]
Rats	Kelulut honey; 200 and 400 mg/kg/day/2 months	↑BV/TV	[93]
		↑Tb.N	
		↓Tb.Sp	
		↑SOD activity ↓MDA activity	
Rats	Kelulut honey	↓Oc.S/BS	[94]
	1 g/kg/8 weeks	↓OS/BS	
Rats	Apis melifera honey; 1, 2, and 4 g/kg/2 weeks	↑ALP	[95]
Bone cells Rat/HC-induced bone damage	Greek thyme + honey	↓ROS ↓Lipid peroxidation Synergistic improving effect on parameters of bone turnover	[96]
Postmenopausal women (*n* = 39)	Tualang honey; 20 mg/day/4 months	No difference in BMD and cardiovascular risk between honey and HRT groups	[88]
Postmenopausal women (*n* = 78)	Tualang honey; 20 g/day/16 weeks	↓Blood oxidative stress	[89]

ALP—alkaline phosphatase; HRT—hormone replacement therapy; BMD—bone mineral density; BV/TV—bone volume per tissue volume; MDA—malondialdehyde; Oc.S/BS—osteoclast surface/bone surface; OS/BS—osteoid surface/bone surface; OVX—ovariectomized; ROS—reactive oxygen species; SOD—superoxide dismutase; Tb.Th—trabecular thickness, Tb.N—trabecular number; Tb.Sp—trabecular separation; ↑—increased; ↓—decreased.

**Table 2:** Preclinical and clinical studies on honey’s potential against breast cancer.

Research Models	Applied Treatment	Obtained Results	References
Cells MCF-7; MDA-MB-231 HeLa	Tualang honey; 1–10%/72 h	↑Cytotoxicity ↑Cell death ↑Apoptosis ↓Δψm ↑Caspase-3/7 and -9	[102]
Cells MCF-7; MDA-MB-231	Tualang honey; 10%/6, 24, 48, and 72 h	↑Apoptosis ↑Caspase-3/7 and -9 ↓TAM-induced adverse effects	[118]
Cells MDA-MB-231; MDA-MB-435; MCF-7	Manuka honey; 0.3–1.25 %/24–72 h	↓Viability of cancer cells ↑Caspase-dependent apoptosis ↑Bax protein expression ↑Apoptosis ↓IL-6/STAT3 signaling pathway	[122]
Cells MCF-7	Greek honey extract	↓Viability of MCF-7 cells	[125]
Cells MCF-7	Fir honey extract 0.2–125 μg/ml	↑Viability of MCF-7 cells	[100]
Cells MCF7, SKBR3, and MDAMB-231	Chestnut, pine, cedar, multifloral honey; 1, 2.5, 5, 7.5, and 10 µg/mL/ 24, 48, and 72 h	↑Cytotoxic effect	[126]
Rats	Tualang honey; 0.2, 1.0, and 2.0 g/kg/day/150 days	↓Tumor development ↓Tumor mean size ↓VEGF protein	[119]
Rats	Tualang honey, Manuka honey 1.0 g/kg/day/120 days	↓Cancer masses ↓Tumor size, weight, and multiplicity ↓Growth rate ↑Expression of pro-apoptotic proteins (Apaf-1, Caspase-9, IFN-γ, IFNGR1, and p53) ↓Expression of anti-apoptotic proteins (TNF-α, COX-2, and Bcl-xL 1)	[124]
Postmenopausal breast cancer women (*n* = 72)	Tualang honey; 20 g/day/12 weeks	↓Alanine aminotransferase levels ↑Creatinine levels ↑Leukocyte counts ↑Platelet counts	[120]
Postmenopausal breast cancer women (*n* = 40)	Tualang honey; 20 g/day/6 months	↓BPE	[121]
Adult women with breast cancer (*n* = 30)	Dorsata honey; 15 mL/3 times daily/15 days	↑IL-3	[127]
Adult women with breast cancer (*n* = 30)	Dorsata honey; 15 mL/3 times daily/15 days	↑T lymphocytes levels No differences in IL-6 level	[107]
Adult women with breast cancer (*n* = 117)	Honey + cinnamon powder; 30 g + 4 g/3 times daily/1 week	↑Overall quality of life	[130]

Δψm—mitochondrial membrane potential; Apaf-1—apoptotic protease activating factor-1; Bcl-xL 1—B-cell lymphoma-extra large; BPE—background parenchymal enhancement; COX-2—cyclooxygenase-2; HeLa—cervical carcinoma; IFN-γ—interferon gamma; IFNGR1—interferon gamma receptor 1; IL-3—interleukin 3; IL-6/STAT3—interleukin-6/tyrosine-phosphorylated; MCF-7, MDA-MB-231, MDA-MB-435—human breast adenocarcinoma cell line; p53—tumor protein; SKBR-3—human breast cancer cell line; STAT3—signal transducer and activator of transcription 3; TAM—tamoxifen; TNF-α—tumor necrosis factor alpha; VEGF—vascular endothelial growth factor; ↑—increased; ↓—decreased.

## Data Availability

Not applicable.
